# Editorial: Exploring diverse personal and social challenges impact on athletes' careers and competition preparation: psychological side effects

**DOI:** 10.3389/fpsyg.2025.1744939

**Published:** 2025-12-17

**Authors:** Ricardo de la Vega, Margarita Limón, Aurelio Olmedilla, Mauricio Garzón

**Affiliations:** 1 Departamento de Educación Física, Deporte y Motricidad Humana, Universidad Autónoma de Madrid, Madrid, Spain; 2 Departamento de Psicología Básica, Universidad Autónoma de Madrid, Madrid, Spain; 3 Universidad de Murcia, Murcia, Spain; 4 Département des Sciences de l'activité physique, Université du Québec à Montréal, Montréal, QC, Canada

**Keywords:** athletes' careers development, athletes social challenges' impact, athletes' performance under pressure, athletes' career transitions, athletes' challenges psychological side effects

## Introduction

Athletes' careers are characterized by continuous transitions and challenges that extend far beyond the boundaries of training and competition. The modern athlete is exposed not only to performance-related stress but also to a wide spectrum of social, cultural, and personal circumstances that shape psychological functioning and influence the capacity to perform under pressure. Situations such as migration, war, economic instability, or major personal life events introduce significant changes in daily routines, identity construction, and emotional regulation. Understanding how these challenges affect athletes' cognition, emotion, and behavior has become a fundamental task in contemporary sport psychology.

This Research Topic, “*Exploring diverse personal and social challenges impact on athletes*' *careers and competition preparation: psychological side effects*,” was conceived as an effort to integrate theoretical, empirical, and applied research addressing the psychological consequences of social and personal transitions in athletes. The aim was to provide a platform for international researchers to discuss how life circumstances intersect with performance preparation, how athletes manage uncertainty and stress, and how psychological interventions can mitigate negative outcomes. By focusing on cognitive, emotional, psycho-physiological, and behavioral dimensions, this collection offers a comprehensive exploration of the human side of high-level sport.

The contributions gathered in this issue reflect a variety of approaches—from experimental studies and psychometric validation to systematic review and meta-analysis—and encompass athletes of different levels, from student-athletes to elite professionals. Together, they contribute to a growing body of evidence demonstrating that athletic performance cannot be understood in isolation from the broader social context and the internal psychological mechanisms that regulate adaptation to change. The studies included provide new perspectives on topics such as stress, resilience, mindfulness, attributional style, competitive orientation, self-control, and decision-making under pressure.

## Contributions

The study by da Cruz et al. examined the psychological effects of social isolation on elite soccer players during the COVID-19 lockdown, with a specific focus on anxiety and sleep quality. Using a longitudinal design that compared data from pre-pandemic training to lockdown conditions, the researchers analyzed responses from 76 professional athletes from Brazilian clubs. Measures of anxiety, sleep quality, and daytime sleepiness revealed significant increases in anxiety during confinement, while sleep patterns and daytime sleepiness remained largely unaffected. These findings suggest that the abrupt disruption of daily training routines and social interactions amplified emotional distress, even among athletes accustomed to coping with stress.

The authors emphasized that although sleep quality was relatively preserved, the emotional toll of isolation should not be underestimated. Anxiety was likely influenced by uncertainty about competition schedules, physical deconditioning, and the loss of team cohesion. The study adds valuable longitudinal evidence to the literature on psychological adaptation during crisis periods, highlighting the role of structured routines and psychological monitoring as protective factors. Importantly, it reinforces the need to include mental health assessment within standard performance-tracking systems in elite sport.

Beyond the immediate implications for pandemic-related confinement, this research also underscores broader themes relevant to the Research Topic: How social isolation, whether due to injury, migration, or other disruptions, can affect athletes' psychological equilibrium. The capacity to maintain emotional stability amid external uncertainty remains a key determinant of resilience and long-term performance consistency.

Huang et al. investigated the role of attribution training as a psychological intervention to mitigate the effects of choking under pressure among elite athletes. The study recruited 328 athletes from Central China and employed a mixed-methods approach combining online and offline questionnaires. Attribution training was designed to modify maladaptive explanations for failure, reduce fear of failure and self-criticism, and enhance self-efficacy. The results showed significant reductions in negative emotional variables and substantial improvements in confidence and perceived competence after the intervention.

The findings provide compelling empirical support for the use of attributional retraining programs to prevent performance deterioration under pressure. Athletes who learned to attribute failure to controllable and unstable causes demonstrated improved coping and reduced avoidance behaviors. This aligns with the theoretical framework of achievement motivation, which posits that the interpretation of success and failure determines persistence and motivation (e.g., Nicholls, 1992; Roberts and Papaioannou, 2014; Stefanek and Peters, 2011). By fostering adaptive attributional patterns, athletes were able to reinterpret setbacks as opportunities for growth rather than threats to self-worth.

This contribution also highlights the practical relevance of psychological education in competitive contexts. Attribution training represents a low-cost, scalable strategy that can be integrated into coaching programs to enhance mental readiness. The study thus bridges applied sport psychology and educational interventions, emphasizing that emotional regulation and cognitive reframing are central to maintaining optimal functioning under pressure.

Johles et al. explored the effects of mindfulness on the quality of life of student-athletes, a population often balancing the dual demands of academic and athletic performance. Through a randomized design involving 99 participants, the authors compared a 4-week body scan intervention with an active relaxation control condition. Body scan intervention is a mindfulness meditation technique that consists in directing attention systematically to the different parts of the body to become aware of the physical sensations present, without judging them. Its main goal is to increase the connection between mind and body to promote deep relaxation, reduce stress and anxiety, and improve overall wellbeing. Active relaxation techniques include deep breathing and progressive muscle relaxation. Measures were collected at baseline, 4 weeks, and 8 weeks to assess changes in five mindfulness facets—acting with awareness, describing, non-judgment, non-reactivity, and observing—as potential mediators of quality of life.

Although the study did not find significant direct effects of the body scan on quality of life or mindfulness facets, it provides critical methodological insights for future interventions. The absence of immediate measurable effects suggests that mindfulness benefits may require longer or more intensive programs, particularly when participants are facing simultaneous academic and athletic stressors (see, for example, Sappington and Longshore, 2015, and more recently, Noetel et al., 2017; Wang et al., 2023). Moreover, the inclusion of an active control condition strengthens the validity of the conclusions by distinguishing between general relaxation effects and specific mindfulness mechanisms.

The authors' contribution extends beyond statistical outcomes by emphasizing the importance of process evaluation in psychological interventions. Their work advocates for a nuanced understanding of how mindfulness interacts with self-regulation, recovery, and wellbeing in developing athletes. It invites researchers to refine both measurement tools and delivery formats to maximize ecological validity in real-world sport and educational settings.

Chen et al. presented a psychometric validation of the Chinese version of the Contesting Orientations Scale (COS) among college athletes. This instrument, originally developed to capture dual orientations toward competition—“partnership” vs. “war”—was adapted and tested within the cultural and linguistic context of Chinese undergraduate athletes. Results demonstrated satisfactory reliability and validity, confirming the two-dimensional structure of the scale and its correlation with established measures of competitive psychology.

The authors' work contributes significantly to the cross-cultural understanding of competition as both a cooperative and adversarial process. The “partnership” orientation frames competition as mutual development and shared pursuit of excellence, while the “war” orientation reflects a more zero-sum, antagonistic mindset. By quantifying these orientations, the scale enables more precise analyses of how cultural norms and moral frameworks influence competitive behavior and interpersonal dynamics in sport.

Beyond psychometrics, the study raises profound conceptual questions about how athletes internalize the meaning of competition. Recognizing the coexistence of cooperative and conflictual motives provides a richer theoretical basis for understanding motivation, ethics, and sportsmanship. Future research could explore how these orientations evolve across career stages and how they relate to outcomes such as resilience, empathy, and long-term engagement in sport.

Yan et al. conducted a systematic meta-analysis to quantify the effects of ego depletion on sports performance. Drawing from eleven articles and twelve experimental studies, the authors calculated an overall moderate negative effect size, indicating that ego depletion significantly reduces athletic performance. Subgroup analyses revealed that the Stroop task generated stronger depletion effects than transcription tasks, and that precision-based skills were more affected than endurance-based activities.

The review synthesizes a decade of research on self-control and performance, offering quantitative evidence that supports the self-control strength model. The findings emphasize that self-regulatory resources are limited and that their depletion can compromise motor execution, focus, and decision-making in competitive contexts. The differentiation across task types provides new insights into the situational vulnerability of athletes to cognitive fatigue and highlights the importance of recovery protocols (Skala and Zemková, 2022).

The implications of this meta-analysis extend to applied sport psychology and coaching. Training programs should not only target physical conditioning, but also the management of self-control resources. Interventions such as mental recovery sessions, attentional training, and deliberate rest may buffer the effects of ego depletion, fostering sustainable high performance. The review thus bridges experimental psychology and applied practice, positioning ego depletion as a central variable in performance science.

The study by Shangguan and Zha investigated the combined effects of framing, competitive state, and time pressure on risk-taking behavior in tennis players across different skill levels. Using an experimental design involving 120 players categorized as novice, skilled, and expert, the authors examined how decision-making was shaped by contextual framing (gain vs. loss), competitive situation (leading vs. trailing), and temporal constraints.

The results revealed distinct patterns of decision-making across expertise levels. Novices were most influenced by framing effects, showing greater susceptibility to emotional and contextual cues. Skilled players demonstrated transitional behavior—sensitive to framing under low pressure but conservative under time constraints—while experts exhibited stable decision strategies, modulated primarily by the competitive state rather than by framing or time pressure. Dual theories of thinking (e.g., Wason and Evans, 1975; Evans, 2008; Evans and Stanovich, 2013; Kahneman, 2011) offer a framework to interpret Shangguan and Zha' results, illustrating the need for further research for understanding how type 1 (automatic, based often on intuition) and type 2 ways of processing interact in sports performance.

These findings deepen understanding of cognitive adaptability in sport. The progression from context-dependent to context-independent decision-making reflects the development of expertise as athletes learn to prioritize task-relevant cues over emotional bias. This study provides valuable evidence to the literature on cognitive control, risk assessment, and performance consistency, with direct implications for training decision-making skills in sport environments.

## Summary

Collectively, the articles in this Research Topic illuminate the complex interplay between psychological, cognitive, and social dimensions of sport. From the effects of social isolation and emotional regulation to decision-making and self-control, these studies illustrate how personal and environmental challenges shape athletes' preparation and performance. They reaffirm the necessity of adopting an integrative approach that accounts for emotional, motivational, and cultural variables in understanding sport behavior.

Across methodologies, the convergence of findings points toward a shared conclusion: psychological resilience and adaptability are not static traits, but dynamic processes shaped by experience, context, and intervention. Interventions such as attributional retraining, mindfulness practice, and structured recovery strategies offer promising pathways to strengthen these processes. Moreover, the inclusion of psychometric and meta-analytic studies underscores the field's movement toward methodological rigor and cross-cultural generalization.

Future research should continue to explore how athletes navigate periods of transition and adversity, integrating physiological, behavioral, and contextual data. By broadening the focus from performance outcomes to holistic wellbeing, sport psychology can better support athletes in managing the multiple identities and demands inherent in their careers. Thus, this Research Topic contributes to a more human-centered understanding of performance, where excellence and health are viewed as complementary rather than competing goals.

## References

[B1] Evans J. (2008). Dual-processing accounts of reasoning, judgment, and social cognition. Annu. Rev. Psychol. 59, 255–278. doi: 10.1146/annurev.psych.59.103006.093629 18154502

[B2] Evans J. S. B. T. Stanovich K. E. (2013). Dual-process theories of higher cognition: advancing the debate. Perspect. Psychol. Sci. 8:223241. doi: 10.1177/1745691612460685 26172965

[B3] Kahneman D. (2011). Thinking, Fast and Slow. New York, NY: Farrar, Straus and Giroux.

[B4] Nicholls J. G. (1992). “The general and the specific in the development and expression of achievement motivation,” in Motivation in Sport and Exercise, ed. G. Roberts (Human Kinetics.), 31–56.

[B5] Noetel M. Ciarrochi J. Van Zanden B. Lonsdale C. (2017). Mindfulness and acceptance approaches to sporting performance enhancement: a systematic review. Int. Rev. Sport Exerc. Psychol. 12, 139–175. doi: 10.1080/1750984X.2017.1387803

[B6] Roberts G. C. Papaioannou A. G. (2014). “Achievement motivation in sport settings,” in Routledge Companion to Sport and Exercise Psychology: Global Perspectives and Fundamental Concepts, eds. A. G. Papaioannou and D. Hackfort (London: Routledge; Taylor and Francis Group), 49–66.

[B7] Sappington R. Longshore K. (2015). Systematically reviewing the efficacy of mindfulness-based interventions for enhanced athletic performance. J. Clin. Sport Psychol. 9, 232–262. doi: 10.1123/jcsp.2014-0017

[B8] Skala F. Zemková E. (2022). Effects of acute fatigue on cognitive performance in team sport players: does it change the way they perform? A scoping review. Appl. Sci. 12:1736. doi: 10.3390/app12031736

[B9] Stefanek K. A. Peters H. J. (2011). “Motivation in sport: theory and application,” in Handbook of Motivational Counseling: Goal-Based Approaches to Assessment and Intervention With Addiction and Other Problems, 2nd Edn.,eds. W. M. Cox and E. Klinger (Oxford: Wiley Blackwell), 415–435. doi: 10.1002/9780470979952.ch17

[B10] Wang Y. Lei S. M. Fan J. (2023). Effects of mindfulness-based interventions on promoting athletic performance and related factors among athletes: a systematic review and meta-analysis of randomized controlled trial. Int. J. Environ. Res. Public Health 20:2038. doi: 10.3390/ijerph20032038 36767403 PMC9915077

[B11] Wason P. C. Evans J. S. B. T. (1975). Dual processes in reasoning? Cognition 3, 141–154. doi: 10.1016/0010-0277(74)90017-1

